# Opportunity cost estimates for spatial conservation prioritisation across terrestrial Europe

**DOI:** 10.1038/s41597-025-05837-5

**Published:** 2025-09-29

**Authors:** Douglas Spencer, Alexandra Marques, Clara J. Veerkamp, Martijn van der Marel, Peter Verburg, Moreno Di Marco, Martin Jung, Heini Kujala, Louise O’Connor, Piero Visconti, Aafke M. Schipper

**Affiliations:** 1 PBL Netherlands Environmental Assessment Agency, Global Sustainability, The Hague, The Netherlands; 2 Institute for Environmental Studies, Vrije Universiteit Amsterdam, Amsterdam, The Netherlands; 3 Department of Biology and Biotechnologies Charles Darwin, Sapienza University of Rome, Rome, Italy; 4 Biodiversity, Ecology, and Conservation Research Group, International Institute for Applied Systems Analysis (IIASA), Laxenburg, Austria; 5 Finnish Museum of Natural History, University of Helsinki, Helsinki, Finland; 6 Radboud University, Radboud Institute for Biological and Environmental Sciences (RIBES), Department of Environmental Science, Nijmegen, The Netherlands

**Keywords:** Environmental economics, Conservation biology

## Abstract

Opportunity costs of conservation represent the foregone economic benefits from using land for nature conservation rather than alternative activities. Considering opportunity costs therefore helps in identifying cost-effective solutions in conservation prioritisation analyses. Since comprehensive, high-resolution, pan-European opportunity cost data are currently lacking, we created a 1 km European layer of opportunity costs (€/ha/yr) for arable, pastoral, forestry and urban lands. To create this layer, we estimated the opportunity costs of productive lands (agriculture and forestry) based on (sub)national land and resource rent data, which we allocated to the grid level integrating agricultural and forestry yield data with country-specific commodity prices. We estimated opportunity costs of urban land based on area-standardised residential rents and urban population density using empirical data from 42 European residential areas. Across land types, urban land comes with the highest opportunity costs, followed by arable, pastoral, and forestry land. We envisage this new opportunity cost layer to be particular useful for broad-scale European conservation prioritisation analyses.

## Background & Summary

Biodiversity is disappearing at unusually fast rates, often considered to be reflecting an ongoing sixth mass extinction^1^. Humans threaten biodiversity through a multitude of activities, including pollution, introducing invasive alien species, over-exploitation of natural resources, and conversion and fragmentation of natural habitat^2^. This biodiversity crisis has prompted various international agreements to halt or reverse the loss, among others by expanding and effectively managing protected areas (PA) to reduce and avert further species decline^3,4^. In Europe, the development of a coherent protected area network is an integral part of the EU Biodiversity Strategy, with the aim to protect 30% of European land, of which one third being under strict protection^5^.

Spatial conservation prioritisation is commonly used to identify priority sites for the implementation of one or more conservation actions. Spatial conservation prioritisation typically aims to identify those sites where biodiversity and ecosystem services conservation can be achieved in a cost-efficient way, i.e. at minimum cost^6,7^. In this context, resource use efficiency is commonly approximated by minimising the total number of sites or total area assigned for conservation, which comes with the implicit assumption that the costs of conservation are the same everywhere^8,9^. This simplification may reflect, among others, a lack of appropriate cost data, reluctance to add further complexity to prioritisation efforts, or a lack of trust in the reliability of the cost estimates^10,11^. For example, a recent literature review revealed that out of 226 studies that use a systematic conservation prioritisation or related approach, only 42 considered costs^12^. Particularly across larger areas (e.g., continental or global extent), comprehensive and unified cost data is often not available. Hence, analyses often resort to proxies, such as land area or agricultural gross revenue, to capture the magnitude and spatial heterogeneity of conservation costs^13–15^. To allow for nuances in the allocation of conservation priorities beyond spatial patterns of biodiversity, spatially-explicit cost estimates can offer an important additional input^13,14,16,17^.

Conservation costs typically include five main components: acquisition costs, management costs, damage costs, transaction costs, and opportunity costs^8^. A comprehensive accounting of costs would include all these components, but obtaining all required data remains difficult or impossible^9,18^. For example, data on the purchase prices of land are typically unavailable across large spatial extents^13^. This may explain why large-scale spatial conservation prioritisations often rely on estimates or proxies of opportunity costs: the foregone economic benefits from using land for alternative activities^8,9^. For example, Naidoo and Iwamura^13^ established a global map of opportunity costs of agricultural lands by combining spatial data on crops and livestock with empirical data on crop and meat prices^13^. More recently, Doelman *et al*.^19^ obtained a global agricultural opportunity cost map by downscaling regional land rent data obtained from a macro-economic model to the grid level, using spatial data on crop yield and crop prices^19^. However, both maps had a relatively low resolution (5 arc-minutes; about 10 km at the equator), and did not include opportunity costs associated with land uses other than agriculture.

**Fig. 1:**
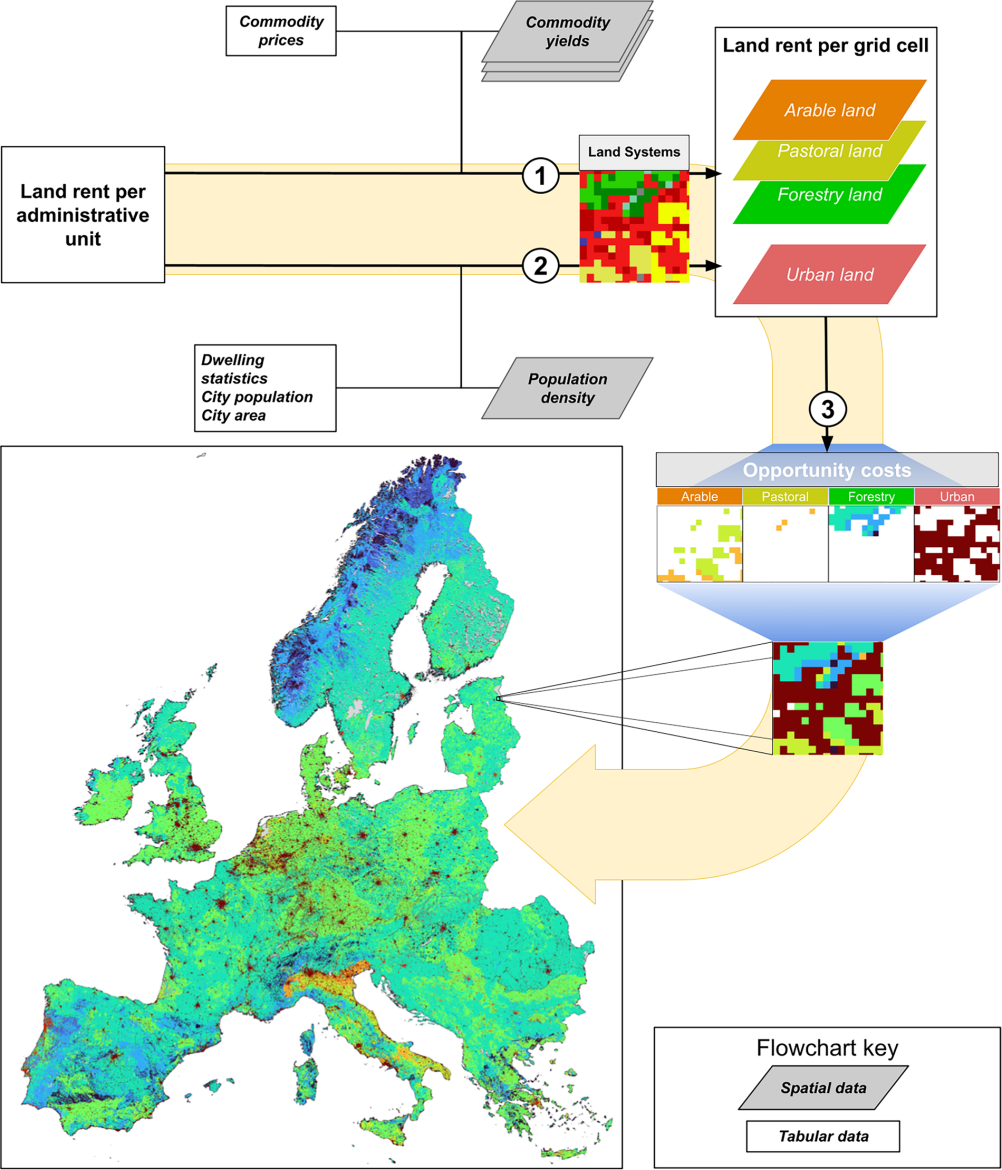
Schematic overview of the procedure to generate the European Land Opportunity Costs of Conservation (EULOCC1k) dataset. (1) Proportional allocation of regional arable, pastoral and forestry land rents. (2) Area-standardisation of residential land rents and extrapolation based on population density. (3) Combination of land type-specific opportunity costs into a single layer.

Here we present the European Land Opportunity Costs of Conservation map at 1 km (EULOCC1K): an area-standardised pan-European opportunity cost map, including both productive lands (agriculture and forestry) and urban land^20^. As a basis, we use a European land systems map at a resolution of 1 km^21^. The map distinguishes between nine land systems, which are further subdivided into 20 classes. For our purpose, we reclassified the land systems into six categories: arable land, pastoral land, forestry land, urban land, and unproductive land (wetland, shrubland, bare land and land covered by snow and rocks) (Supplementary Table 1). For arable, pastoral and forestry lands (productive land), we derived opportunity costs from regional and (sub)national land and resource rent values, which we allocate to the grid level based on crop, livestock and wood yield maps combined with national commodity prices (Fig. 1). For urban land, we used residential rents based on empirical data for 42 residential areas across Europe. We extrapolated these across Europe based on a quantitative relationship between residential rent and urban population density. We then combined the four opportunity cost layers to form EULOCC1K, with units of annual rent per unit of area (€/ha/yr). We assume that the opportunity costs of conserving economically unproductive natural lands (wetland, shrubland, bare land and land covered by snow, ice and rocks) are negligible and set these to zero. To cancel out or reduce inherent macro-economic differences between countries, we also provide Z-score and Purchasing Power Parity standardised layers.

## Methods

### Opportunity costs of productive land

#### Allocation procedure

For productive lands (i.e., agricultural land, including arable and pastural land, and forestry land), land rent data is available from the Nomenclature of Territorial Units for Statistics (NUTS) level 3 (sub-national administrative regions, like provinces) to level 0 (nations), depending on the country (for a breakdown of land rent data, see Table 1; for information on all input data sources, see Supplementary Table 2). We allocated the coarse-grain land rent data to the grid cell level of the land systems map (1 km) following the approach as described by Doelman *et al*. and Jantke *et al*.^19,22^. Essentially, we assume that in each grid cell the land rent value is proportional to the yield obtained, as (Eq. 1):

1 $${R}_{i,j}={R}_{j}\cdot \frac{{y}_{i,j}}{{\sum }_{i}^{n}({s}_{i,j}\cdot {y}_{i,j})}$$

where *j* denotes a (sub)national region with land rent data available, *i* denotes a grid cell within *j*, ${R}_{i,j}$ refers to the land rent allocated to a given grid cell in a given region (€/ha/yr), ${y}_{i,j}$ refers to the yield value of a grid cell within the region (€/ha/yr), ${R}_{j}$ denotes the regional land rent (€/ha/yr), and ${s}_{i,j}$ refers to the share of each grid cell in the total area of the same land type within region *j* (dimensionless). Since in our study all grid cells have the same area (1 km), ${s}_{i,j}$ is constant, hence we can equate $\mathop{\sum }\limits_{i}^{n}{(s}_{i,j}\cdot {y}_{i,j})$ with the mean of the gridded yield values ${y}_{i,j}$ within a region *j*. The input data required for the opportunity cost estimation for productive lands include land rent data for arable land, pastoral land and forestry land (i.e., variable ${R}_{j}$ in Eq. 1) and spatially explicit monetary-based yield data for these land categories (i.e., variable ${y}_{i,j}$ in Eq. 1) (see Supplementary Table 2). We obtain the latter by multiplying yield data in biophysical units (kg/ha/yr) by the unit price of the commodity (€/kg/yr). In the following sections we further explain how we obtained and processed the land rent data and the yield data.

**Table 1:** Overview of the agricultural (i.e., arable and pastoral) land rent data sources and resolution.

Agricultural land rent data source	Thematic Resolution	Spatial Resolution	Countries
Eurostat^23^	Arable and pastoral land	NUTS 0	EE, LV, LT, LU, MT, FR
		NUTS 1	UK^a^
		NUTS 2	BG, CZ, DK, EL, ES, IE, HR, IT, HU, NL, AT, PL, SI, SK, FI, RO, SE, NO, UK^b^
Federal Statistical Office of Germany^24^	Arable and pastoral land	NUTS 1	DE
Department of Agriculture, Environment and Rural Affairs (of Northern Ireland) (DAERA)^25^ Statistics for Wales^26^ The Scottish Government^27^	Average of all farm types	NUTS 1	UK^c^
Statistics Portugal^29^	Combination of arable and pastoral land	NUTS 2	PT
Federal Statistical Office of Switzerland^28^	Combination of arable and pastoral land	NUTS 3	CH
MAGNET^19^^ d^	Combination of arable and pastoral land	Central Europe	AL, MK, RS, BA^e^, ME, XK^e^, CY

^a^Denotes England only.

^b^Denotes Scotland only.

^c^Denotes Wales and Northern Ireland.

^d^An overview of MAGNET regions and respective countries is given in Supplementary Table 4.

^e^Since Bosnia and Herzegovina and Kosovo do not have NUTS codes, we use BA as two-letter country code for Bosnia and Herzegovina, and XK for Kosovo.

#### Land rent data

##### Arable and pastoral lands

Agricultural (i.e., arable and pastoral) land rent is defined as the price of renting one hectare of agricultural land during the reference period (a calendar year)^23^. Agricultural land rent represents the foregone income of agricultural land owners if the land is allocated for conservation and agricultural activities are abandoned^13^. For arable and pastoral lands, we obtained land rent data from the European Statistical Office (Eurostat)^23^ where available, and where not, we took values from the offices for national statistics^24–29^. Where we could not obtain agricultural land rent data from the offices for national statistics, we obtained agricultural land rent values from the Modular Applied General Equilibrium Tool (MAGNET)^19^. The resolution of the compiled agricultural land rent values varies from the NUTS 3 level to the supra-national level of the MAGNET regions (Table 1). In the Eurostat dataset, rent data are available for three categories: arable land, permanent grassland, and arable land/permanent grassland, where the latter are an average of the arable and permanent grassland rents^23^. Where available, we used land rent data specific to arable land or pastoral land (using the permanent grassland rents), and where not, we used the combined arable/permanent grassland rent values. The latter applied to 16 countries (AT, CZ, DK, EE, EL, ES, FI, FR, LU, LV, MT, NL, PL, SE, SI, and UK; for an overview of two letter country codes and corresponding countries, see Supplementary Table 3).

Statistics Portugal provides total agricultural land rent values at NUTS 2 level, instead of land rent values per unit area^29^. In the absence of NUTS 2 agricultural area values from Statistics Portugal, we converted these values to rent per hectare values based on the total area of cropland and grassland per NUTS 2 region, obtained from the land systems map. Similarly, the Federal Statistical Office of Switzerland provides the total agricultural land rent values per NUTS 3 region^28^, so we used the total agricultural area that was also provided by the Federal Statistical Office of Switzerland to determine the €/ha value for each NUTS 3 region^30^. For Wales and Northern Ireland, land rent data are available per hectare and per farm type, which does not necessarily conform to the arable-pastoral land split. Therefore, we used the average value across farm types as a combined agricultural land rent value. From the remaining countries with agricultural land rent data from national statistics offices (Scotland and Germany), only Germany had separate values for arable and permanent grassland land rents.

If land rent values were provided in a currency other than euro, we converted them to euros based on the exchange rate specific to the corresponding year. We then converted all land rents to 2021, which was the most recent year available in Eurostat^23^ at the time of analysis (i.e., first half of 2023), using a year-to-year Eurozone-specific annual inflation rate of the euro, referred hereafter as the nominal-to-real conversion^31^.

##### Forestry lands

In Europe, leasing forestry land is far less common than leasing agricultural land^32^, and so it is more appropriate to use the net economic benefits of forestry (profits or revenues minus production costs) as the proxy of opportunity cost of conservation in forestry land. We obtained forestry land rent data from the World Bank^33^, which provides rent data at the national level (i.e., NUTS 0) as a percentage of a country’s GDP for domestic timber and non-timber forestry production per year. We use the World Bank’s GDP data^34^ to convert the relative values to absolute monetary values for the year 2021 and then convert these values into a per hectare unit using the total area of wooded land per country according to Eurostat^35^. We took all values from 2021, and like the agricultural land rent data, we converted non-euro currencies to euros. The forestry rent dataset covers all European countries except six (i.e., Andorra (AD), Liechtenstein (LI), Kosovo (XK), Malta (MT), San Marino (SM) and the Vatican City (VA)). For countries without forestry rent values yet containing forest land according to the land systems map^36^, we used the average forestry land rent across all neighbouring countries with data available. We defined neighbours as countries sharing terrestrial or maritime borders, as follows: for Andorra, ES and FR; for Liechtenstein, AT and CH; for Malta, EL and IT; for San Marino, IT; for Kosovo, AL, RS and MK (see Supplementary Table 3 for country codes).

#### Yield data

##### Arable land

To obtain yield data for arable land, we combined gridded crop yield data (kg/ha/yr) with the country-specific unit price per crop (€/kg). We obtained crop yields from the Spatial Production Allocation Model 2010 (MapSPAM) (v2) dataset^37,38^, which provides spatially explicit yield data at a 5 arc-minute resolution (approximately 10 km at the equator) for 42 crop categories. We included all MapSPAM crop categories that are produced in Europe, according to The Food and Agriculture Organization Corporate Statistical Database (FAOSTAT)^39^ and MapSPAM^37,38^, and with crop price data available (Supplementary Table 5). We used the total crop yield per cell (kg/ha/yr) across all farming technologies included in MapSPAM (irrigated, rainfed high inputs, rainfed low inputs, rainfed subsistence and rainfed), obtained as harvested area weighted average of the four farming technology yields^37^. We resampled the MapSPAM layers to the projection and resolution of the land systems map (ETRS LAEA EPSG:3035, 1 km), using nearest neighbour and bilinear resampling, respectively.

We obtained crop producer prices for the years 2001 to 2021 from Eurostat^40^ and FAOSTAT^41^, available at the country (NUTS 0) level. Primarily, we used the crop producer prices from Eurostat (€/t)^40^, either for the year 2021 when available or for the first available preceding year. In absence of producer prices in Eurostat, we took country-level FAOSTAT producer prices^41^, which are provided in US dollars per tonne (USD/t). Crop price data were missing for three countries (CY, XK and ME). For these countries we used an average of the prices of neighbouring countries (i.e., for Kosovo from RS, AL and MK; for Montenegro from BA, RS, and AL; and for Cyprus from EL, since this is the only European country sharing a (maritime) border with CY). Similar to the land rent data, we first converted non-euro currencies to euros and then standardised all values to 2021 levels using year-to-year inflation rates^31^. Finally, we converted prices per tonne to prices per kg for consistency with the MapSPAM data.

To combine the yield and the price data, we matched the MapSPAM crop categories, which are based on the FAOSTAT system of crop categorisation, with the Eurostat system of crop categorisation (Supplementary Table 5)^42^. For MapSPAM crop categories that correspond to multiple crops in the crop price dataset, such as temperate fruit, we averaged the prices across the corresponding crops. Finally, we averaged the monetary yield values across the crop categories present in a grid cell to obtain a single monetary crop yield value per grid cell, as (Eq. 2):

2 $${y}_{a,i,j}=\frac{{\sum }_{1}^{c}{y}_{c,i,j}\cdot {p}_{c,j}}{{n}_{c,j}}$$

where ${y}_{a,i,j}$ is the monetary yield of arable land in grid cell *i* in region *j* (€/ha/yr), ${y}_{c,i,j}$ is the yield of crop category *c* in grid cell *i* in region *j* (kg/ha/yr), ${p}_{c,j}\,$ is the unit price of crop category *c* in country *j* (€/kg) and ${n}_{c,j}$ is the number of crop categories *c* present in country *j* according to MapSPAM and where price data is available according to the Eurostat^40^ and FAOSTAT^41^.

##### Pastoral land

We quantify the yield of pastoral land based on livestock production. Since livestock production data is only available at the country level (NUTS 0)^43,44^, we used the livestock density data from the Gridded Livestock of the World (GLW4) database (year 2015)^45–48^ to account for within-county variability. We assumed that pastoral land is occupied primarily by grazing animals and therefore selected grazing ruminant livestock from the GLW4 dataset: cattle, goats, and sheep. We excluded horses assuming their contribution to livestock products (meat and milk) is negligible. While reindeer are an important source of meat and milk in Nordic countries, we could not include them because they are absent from the GLW4 data. In addition, reindeer are typically allowed to roam freely, including in protected areas, hence setting aside land for nature conservation does not come with opportunity costs^49^. The GLW4 dataset has a spatial resolution of 5 arc-minutes (approximately 10 km at the equator) and includes dasymetric and areal-weighted livestock density maps (expressed in heads per 10 km) (see Supplementary Table 2 for all pastoral land inputs). We selected the dasymetric version because of its more realistic distribution patterns^45^. Since the GLW4 maps are in the same projection and resolution as the MapSPAM maps, we took the same approach to resample the maps to align the resolution and projection to the land systems map. We then divided the result by 1,000 to convert the units from heads per 10 km to heads per hectare, assuming homogenous distribution of livestock with the larger 10 km grid cell.

Next, we estimated the yearly monetary value per head of cattle, sheep and goats (€/head/yr). To that end, we quantified the total monetary yield per livestock species per region per year, and divided this by the total number of animals of that species per region, as (Eq. 3):

3 $${y}_{l,j}=\frac{{\sum }_{1}^{q}{y}_{q,l,j}\cdot {p}_{q,l,j}}{{\sum }_{1}^{i}{n}_{l,i,j}}$$

where ${y}_{l,j}$ is the monetary yield of livestock species *l* in region *j* (€/head/yr), ${y}_{p,l,j}$ is the yield of product *q* (milk or meat) obtained from livestock species *l* in region *j* (in kg/yr), ${p}_{q,l,j}$ is the unit price of product *q* (milk or meat) obtained from livestock species *l* in region *j* (in €/kg), and ${n}_{l,i,j}$ is the number of animals of livestock species *l* in cell *i* in region *j* (heads), as obtained from the GLW4 data.

We obtained the total annual production estimates of milk and meat per livestock species per country from Eurostat^43,44^, which were available for nearly all countries (except Kosovo). Where 2021 values of production were not available, we took the value from the most recent year reported. We obtained unit producer prices (€/kg) from Eurostat^50^ and FAOSTAT^41^ for the meat and milk of cattle, sheep, and goats. We note that the availability of price data was higher for cattle than for goats and sheep. We missed producer prices for all livestock species from Montenegro, Serbia, and North Macedonia, and since these countries neighbour each other, we took an average of the producer prices across the countries in the Central Europe MAGNET region. For Kosovo, we missed both production and price data, and we used the livestock-specific €/head value from Albania, which was the only neighbouring country with price and production data. San Marino and Andorra also missed price and production data, and therefore we used the Italian values and an average of the French and Spanish values, respectively.

Next, we multiplied the monetary yield per head values with the GLW4 gridded population maps to obtain gridded livestock monetary yield maps per livestock species (€/ha/yr), as (Eq. 4):

4 $${y}_{l,i,j}={y}_{l,j}\cdot {d}_{l,i,j}$$

where ${y}_{l,i,j}$ is the monetary yield of livestock species *l* in grid cell *i* in region *j* (€/ha/yr), ${y}_{l,j}$ is the per capita yield of livestock species *l* in region *j* (€/head/yr) as obtained with Eq. 3, ${d}_{l,i,j}$ is the population density of livestock species *l* in grid cell *i* in region *j* (heads/ha) as obtained from the GLW4 map. Finally, we averaged the yield data across the livestock species present in a grid cell to arrive at the total monetary pastoral yield per grid cell. We excluded a species if there was no data on commodity prices.

##### Forestry land

We obtained timber yield values in monetary terms by multiplying grid-specific woody biomass yield values (available in 1000m^3^/km/yr) by the export timber prices per country (available in €/1000m^3^), as (Eq. 5):

5 $${y}_{f,i,j}=\frac{{y}_{t,i,j}\cdot {p}_{t,j}}{100}$$

where ${y}_{f,i,j}$ is the monetary yield of forestry land in grid cell *i* in region *j* (€/ha/yr), ${y}_{t,i,j}$ is the monetary timber yield in grid cell *i* in region *j* (1000m^3^/km/yr), ${p}_{t,j}$ is the unit price of timber-based product (roundwood, sawnwood, wood pulp or a weighted mean of sawnwood and wood pulp based on national production outputs) in country *j* (€/1000m^3^), and the division by 100 is to convert from km to ha.

We retrieved gridded woody biomass yield data from the wood production dataset by Verkerk *et al*.^51,52^. They downscaled national wood production statistics (years 2000–2010) for 29 European countries to a 1 km resolution using a regression model that estimates forest yield based on various location characteristics, including climate (precipitation, temperature), terrain ruggedness, productivity and tree species composition. We used the latest map available in the dataset, i.e., the annual wood production of 2010, which is in the ETRS89-extended LAEA projection. The projection and resolution of this map are already aligned to our land systems map. The wood production map omits Malta and the Western Balkan states (HR, RS, ME, MK, BA, AL, XK). For the omitted Western Balkan countries, we used average wood production values based on the other nations that are in the MAGNET Central Europe region, and for Malta we took the value from IT.

We obtained roundwood, sawnwood and wood pulp export timber prices (2020USD/1000m^3^) from UNECE/FAO^53^, and converted these into 2021 euro values based on the 2020USD to 2020EUR exchange rate and the inflation rate of the euro from 2020 to 2021. Preferably we used both sawnwood and wood pulp price data, and combined these values using the ratio of the respective wood product type (“Sawlogs and veneer logs” and “Pulpwood, round and split”) removals reported by Eurostat^54^ as the weights. However, if either price were not available, we used roundwood prices as a substitute, as both sawnwood and wood pulp come under the umbrella of roundwood according to the FAOSTAT Forest Product Production Statistics^55^. If roundwood export prices were not available, but either one of sawnwood or wood pulp prices were available, we used the available one as the wood product price. Finally, if no prices were available, we substituted the export prices based on averages across neighbouring countries. Wood price data were missing for six countries (AD, CY, LI, XK, MT and MK). We used the following neighbouring country(s) average: for Andorra, FR and ES; for Cyprus, EL; for Liechtenstein, AT and CH; for North Macedonia, AL, BG, RS, EL; for Malta, IT; for SM, IT; for Kosovo, RS, AL and ME. We preferred to use wood product prices that are representative of the type of wood products that drive production in a region and prices that reflect the earliest stages of the manufacturing process of these products. Pulpwood prices (i.e., for the wood) would have been preferable over wood pulp prices (i.e., for the derived product), but the former were not available in consistent price forms for different countries. Therefore, we chose to use export prices for wood pulp.

### Urban land rent

We approximated opportunity costs for urban land by the rent paid by a tenant to a property owner. We first obtained area-standardised residential rent values available for a sample of European residential areas (n = 42), and then established a linear regression model that relates these rent values with population density and major European region (North, East, South and West, following the regional divisions made by Dou *et al*.^56^) to extrapolate residential rents to all residential areas in the land systems map. The rent dataset includes monthly residential rent values for all capital cities across the European Union and Switzerland for 2021, six additional cities (CH: Genève; IT: Varese; DE: Bonn (2020), Karlsrühe and München) and one village (UK: Culham)^57^. The rent values are specified for different dwelling types: non-detached house, detached house and 1-, 2-, and 3-bedroom flats. As for all monetary-based datasets used in this study. We then converted the monthly values into yearly values to get the annual rent per urban area per dwelling type (€/yr). Converting these rents into per-hectare values requires information on the total number of each dwelling type per urban area. As this information was not available, we made an estimate based on the total population per urban area, the proportion of its population per dwelling type, and the typical household size, as (Eq. 6):

6 $${N}_{d,m}=\frac{{f}_{d,m}\cdot {P}_{m}}{{H}_{d}}$$

where ${N}_{d,m}$ is the number of units per dwelling type *d* in urban area *m*, *P*_*c*_ is the total population in urban area *m*, ${f}_{d,{m}}$ is the fraction of the population in dwelling type *d* in urban area *m*, and ${H}_{d}$ is the typical household size in dwelling type *d*.

We obtained the total population per urban area from Eurostat^58^, UN statistics^59^ and national and municipal statistics offices^59–68^. If available we preferred Eurostat data, thereafter data from UN statistics and again thereafter data from national and municipal statistics offices. Total population values were available for different years, ranging from 2011 to 2017; we took values for the latest year possible. For the proportion of the population in different dwelling types, we used country-level data from Eurostat (2018 to 2021)^58^. Since this dataset distinguishes only houses and flats, we aggregated the dwelling types into these two groups, and averaged the rents. As data on household size was not available, we assumed typical household sizes for houses and flats to be four (a family with two kids) and two (a couple), respectively.

Next, we multiplied the estimated number of units per dwelling type per urban area by the corresponding yearly rent per dwelling type, summed this across dwelling types, and divided by the total size of the urban area to find the total yearly rent per urban area on a per-hectare basis (Eq. 7):

7 $${R}_{m}=\frac{{\sum }_{1}^{d}{r}_{d,m}\cdot {N}_{d,m}}{{A}_{m}}$$

where ${R}_{m}$ is the area-standardized yearly residential rent in urban area *m* (€/ha/yr), ${r}_{d,m}$ is the average yearly rent for dwelling type *d* in urban area *m* (€/yr), ${N}_{d,m}$ is the total number of dwellings of type *d* in urban area *m* (Eq. 6), and *A*_*m*_ is the size (ha) of urban area *m*. We obtained values on size from Eurostat and national and municipal statistical offices^65–67,69–74^.

We then fit a regression model relating the area-standardised yearly rents to the population densities of the residential areas per major European region (north, east, south and west), the latter to account for systematic differences in rent among regions. We obtained the population densities by dividing the total population size of each urban area (*P*_*m*_ in Eq. 6) by its area size (*Am* in Eq. 7). We log-transformed the area size and population densities because of their skewed distribution and we tested multiple model variants, selecting the most appropriate model based on the residual plot and on Akaike’s Information Criterion (AIC; see Supplementary Table 6 and Supplementary Figure 1). Finally, we used the regression model to estimate rent values for all residential areas in the land systems map, using as input population density estimates for 2020 that spatially comply with the land systems map^75^.

### Integrating and standardising opportunity cost layers across land types

Finally, we combined the opportunity cost layers for the four land systems into a single layer. We assumed that the opportunity costs of conserving economically unproductive natural lands (land systems map classes are: bare, rock and shrubs; wetlands; water and glacier) are negligible and set these to zero. To assign opportunity costs to mosaic classes (Forest/shrub and cropland mosaic; Forest/shrub and grassland mosaic), which account for roughly 20% of the land systems map, we determined the share of every named land class in each mosaic cell using the high-resolution input of the land systems map, the Corine Land Cover (CLC) map (2018)^36,76^. We first resampled the CLC map from its original 100 m resolution to a 1 km resolution and then determined the percentage cover of each land category within a given mosaic class cell (see Supplementary Table 1 for land categories). We then quantified the opportunity costs of the mosaic classes proportional to the cover of the constituent classes.

**Fig. 2:**
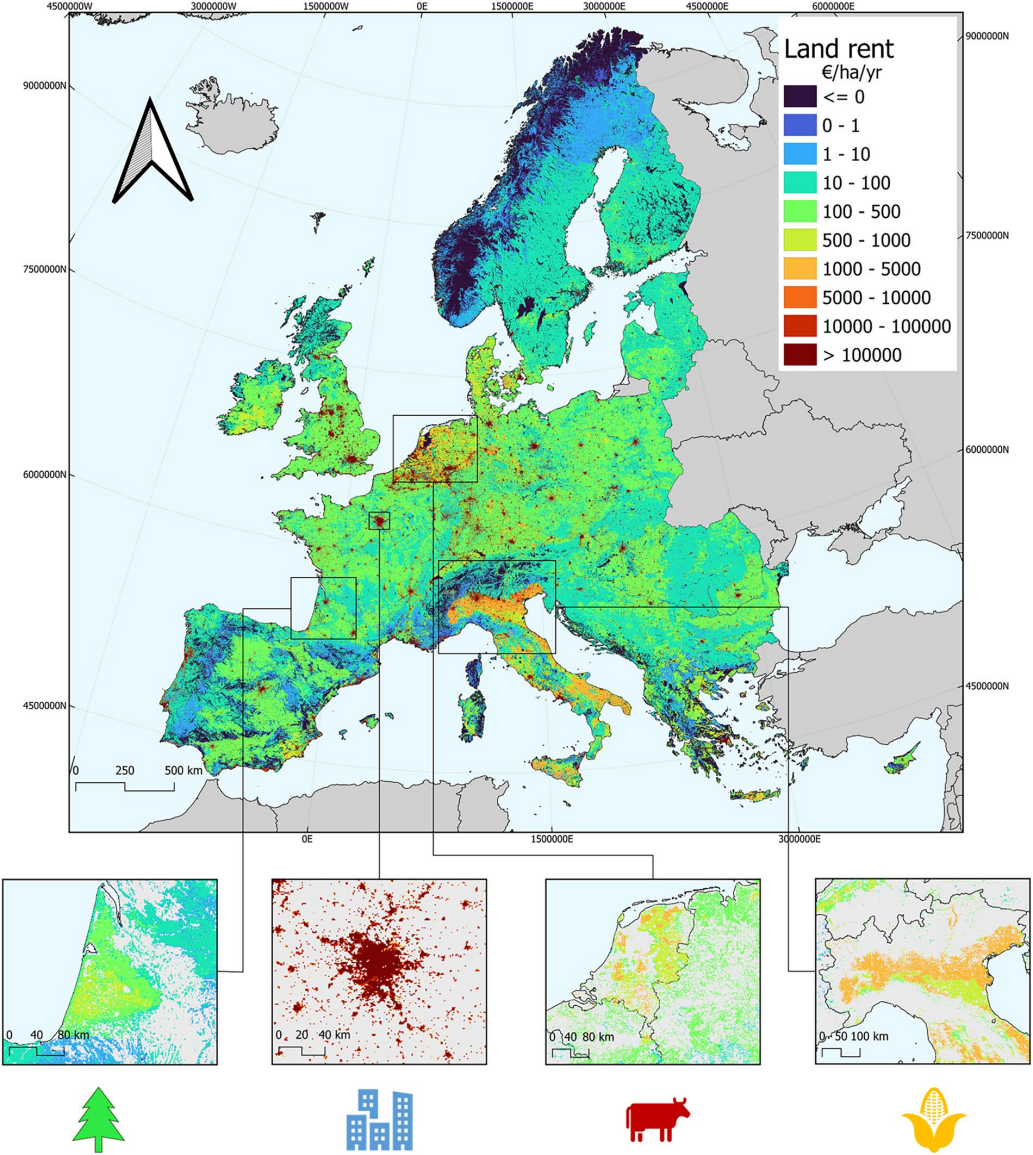
Opportunity costs of conservation across terrestrial Europe. Insets show examples of opportunity costs specific to forestry, urban, pastoral, and arable land (bottom left to bottom right).

The resulting combined layer reveals clear differences in opportunity costs between land types as well as between different regions in Europe (Fig. 2). As this regional variation may bias the results of spatial conservation prioritisation to regions with lower opportunity costs, we also provide two standardised layers. First, we standardised the average opportunity cost values within each country via standard-score (Z-score) normalisation. Second, we standardised the layer based on Eurostat purchasing power parities (PPPs) of national GDPs of each country for 2021^77^. We standardised across countries using the same method as Pirson *et al*.^78^, where we obtain EU-PPP standardised values by dividing the euro estimates by the corresponding PPP values of each country.

## Data Records

The EULOCC1K dataset^20^ contains Europe-wide layers of opportunity costs at a 1 km resolution based on agricultural land, forestry resource or residential rent data (€/ha/yr), representing the foregone economic benefits of agricultural, forestry and residential land when it is allocated for nature conservation or restoration. The EULOCC1K dataset is available as a set of gridded layers. The dataset consists of a combined layer, two standardised layers (PPP and Z-score) and the constituent individual opportunity cost layers, with a georeferenced tagged image file format (GeoTIFF) (Table 2). Layers are in EPSG:3035 - ETRS89-extended/LAEA Europe projection, aligned with the land systems map, and have a resolution of 1 km. The dataset is accessible as a netCDF file on the Essential Biodiversity Variable (EBV) Data Portal repository^20^. A preliminary version of the dataset has been previously described in a publicly available technical report^79^.

**Table 2:** Overview of all map layers that are available with their associated file names and units.

Map layer	File name	Unit
*Opportunity costs for arable land*	Arable_land_opportunity_costs.tif	€/ha/yr
*Opportunity costs for pastoral land*	Pastoral_land_opportunity_costs.tif	€/ha/yr
*Opportunity costs for forestry land*	Forestry_land_opportunity_costs.tif	€/ha/yr
*Opportunity costs for urban land*	Urban_land_opportunity_costs.tif	€/ha/yr
*Combined opportunity cost layer*	Combined_land_opportunity_costs.tif	€/ha/yr
*Purchasing power parity normalised opportunity cost layer*	PPP_standardised_land_opportunity_costs.tif	€/ha/yr
*Z-score normalised opportunity cost layer*	Z_score_standardised_land_opportunity_costs.tif	Dimensionless

Economic data underlying the layers have been standardised to 2021 price levels.

## Technical Validation

**Fig. 3:**
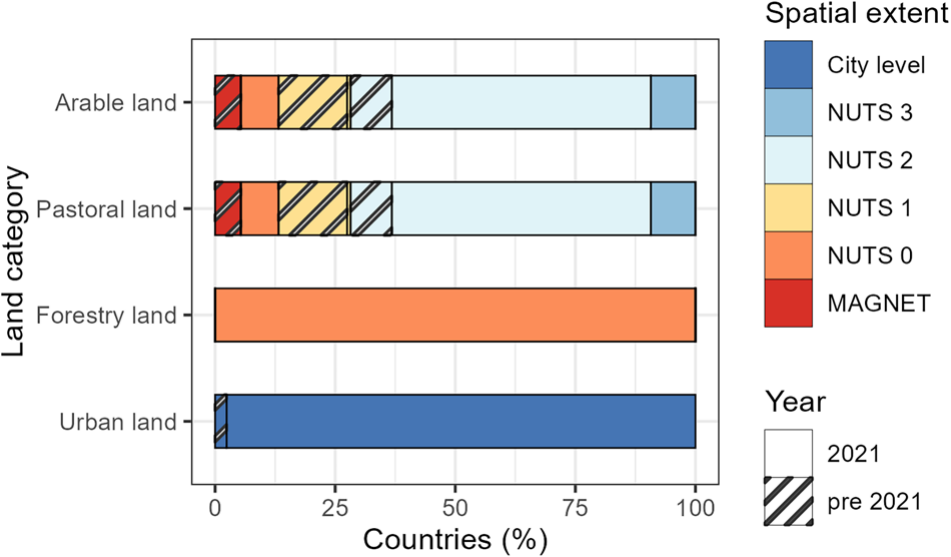
Overview of the spatial resolution of the land rent data.

**Fig. 4:**
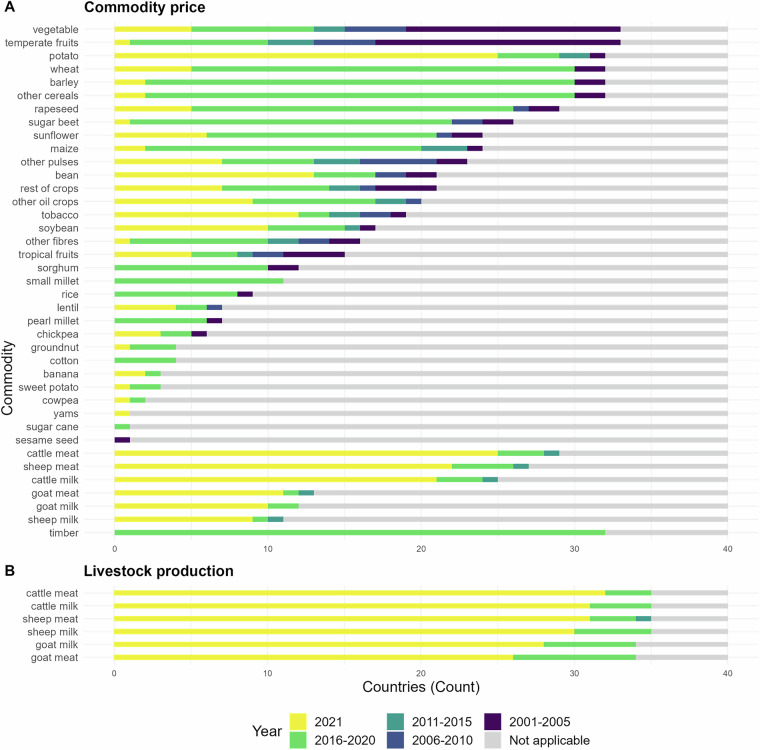
Year of origin of national commodity price (**A**) and livestock production data (**B**).

Since no empirical high-resolution land rent data was readily available to validate the EULOCC1K layer, we performed two quality checks. First, we checked whether the productive land rent allocation (Eq. 1) was implemented correctly by calculating the mean absolute error (MAE) between the mean allocated land rent values across the cells in each region and the original regional input land rent values. If the implementation has been done correctly, the deviation should be negligible. This was indeed the case, as we found an MAE for arable land of 1.74 × 10^−5^ €/ha/yr (n = 315), for the pastoral land of 1.39 × 10^−5^ €/ha/yr (n = 315), and for the forestry land of 4.65 × 10^−6^ €/ha/yr (n = 39). Small deviations between the original regional land rents and the regional average of the allocated rents likely reflects rounding errors due to the downscaling procedure^80^. Second, we evaluated the cost layer in terms of the consistency, spatial resolution, and temporal origin of the various input data sources (Figs. 3, 4). This second evaluation offers users an understanding of where data gaps occur across commodity and land rent data which may help users to decide whether the layers satisfy their needs. For example, in France the spatial resolution of the input agricultural land rent is at the national level, whereas in Switzerland the resolution of the agricultural land rent data is at NUTS 3, hence much more detailed (Table 1).

## Usage Notes

### Scope

It is important to note that the opportunity costs of conservation included in EULOCC1K are representative of the current land use in Europe as represented by the land systems map^36^ underlying the EULOCC1K dataset^20^. Hence, EULOCC1K does not consider potential changes in opportunity costs due to possible future land transitions. For example, the agricultural and forestry opportunity cost layers represent the opportunity costs associated with current agricultural or forestry practices and do not account for foregone profits associated with potential future urban development in agricultural or forestry areas (i.e., option value). Further, we do not consider other types of land use that are not included in the land systems map and that may have significant implications for local land values^81,82^, such as renewable energy infrastructure (e.g., wind or solar parks).

We also note that we refrained from including other economic costs of conservation, such as management and restoration costs^8^, due to inadequate coverage and quality of empirical datasets at the time of analysis. Similarly, we do not account for environmental subsidies, funding and other payments that can support a less intensive use of land, and that users of our dataset may want to consider in prioritisation exercises alongside opportunity cost and other cost estimates. We acknowledge that additional costs as well as economic benefits are important to consider to identify potential trade-offs and synergies between economic activities conducted by local communities and local conservation actions^83^.

### Application

The EULOCC1K dataset is designed to support broad-scale European conservation planning prioritisation efforts. The dataset could be used in combination with layers of biodiversity and ecosystem services to identify priority conservation sites aligned with, for example, the objectives of the EU Biodiversity Strategy for 2030 of protecting 30% of the land for nature^84^, where newly designated protected areas would lead to regulatory restrictions for a range of land uses and extractive activities. We note that optimizing conservation prioritization towards the lowest opportunity costs relies on the implicit assumption that assigning land for conservation purposes will lead the economic activities associated with the current land use to cease. However, certain protected area designations allow for the continuation of low-intensity land uses (e.g. IUCN type VI: Protected areas with sustainable use of natural resources)^85^. Given the spatial resolution and accuracy of the input data sources, we also note that our dataset is better suited for informing conservation planning at national and European-wide scales, rather than for sub-national or local conservation planning. For example, small-scale conservation project in residential areas (e.g., prioritising vacant land for urban nature development) are beyond the applicability domain of our dataset.

To facilitate broad-scale transboundary applications, we provide PPP and Z-score adjusted data that can help to reduce and remove inherent macroeconomic differences between countries. One could use the EU-PPP standardised layer if the aim is to reduce bias between countries due to inherent differences in price levels. The Z-score standardisation allows full removal of any prioritisation bias between countries due to differences in their average land rent values. This Z-score layer is useful in spatial conservation prioritisations if the aim is to allocate the same percentage of land in each country as protected areas.

In addition to opportunity costs and biodiversity and ecosystem features, other relevant constraints and boundary conditions might be useful to consider in the conservation prioritization. For example, EULOCC1K does not include existing protected areas. We suggest using the EULOCC1K map in combination with a protected area map of Europe to account for areas already assigned for nature conservation^86^. Additional information can also be included to account for conservation subsidies (e.g., eco-schemes from the common agricultural policy (CAP) 2023–2027^87^) or for conservation planning constraints that conflict with the land demand for conservation, such as renewable energy facilities or development plans, or land assigned for the development of new residential areas^88^. The latter may alternatively be accounted for by estimating option values, so as to prevent underestimation of opportunity costs of conservation in areas with a high probability of renewable energy or urban development^89^.

### Limitations

Due to the lack of a readily available high resolution land rent dataset that is representative across Europe, users should be aware that we could not validate the allocated land rents with empirical site-specific land rent values. Further, the use of the relatively coarse-grain crop and livestock productivity maps representative of 2010 and 2015 imply that the arable and pastoral land opportunity cost estimates do not reflect fine-grain heterogeneity in yield nor are they necessarily representative of more recent production data. The latter holds also for the forestry production map, which is representative of the year 2010, too. Similar to production, commodity prices as well as differences in prices among commodities change over time, which affects the differences in monetary yield between grid cells hence the allocated rent data.

Whilst we use gridded data on agricultural and forestry production (yield), we acknowledge that the rent and in particular the price data are less granular. While agricultural land rent values are often sub-national (Fig. 3), land rents for forestry are at the national level, and the same holds for the price data. This implies that our opportunity cost layers do not capture fine-grained heterogeneity in rent and prices that may result from, for example, spatial differences in production costs, transport costs due to market access and price differences between crop species or harvested trees. Under the assumption that rent, price and production values are positively correlated^90,91^, the lack of granularity in our rent and price data could lead to an overestimation of spatial heterogeneity in the opportunity cost estimates. In this context, we also mention that the use of regional to national rent data may lead to an overestimation of spatial heterogeneity across region or country borders.

We acknowledge that the opportunity costs of urban land are particularly uncertain. First, our estimates of urban land rent are based on a relatively small sample of rents from predominantly capital cities, where rents might be systematically higher compared to other residential areas^92^, although other factors including tourism and university coexistence may also have a significant effect on rents^93^. Further, our relationship between urban land rent and population density, whilst having a high R^2^ value (0.95), does not account for other within-country differences in urban rent, for example related to regional differences in wealth. In addition, we needed to convert property-based rent values to area-based rent values based on various assumptions (Eq. 6), each associated with uncertainty. In view of these sources of uncertainty, and given the high overall opportunity costs of residential areas in general (Fig. 2), users may want to consider the residential areas in our layer as ‘lost’ and mask them from conservation prioritization exercises, as done in previous studies^94,95^.

We note that we approximate forestry opportunity costs by the net benefits of profit and production, whereas the agricultural and the urban opportunity cost layers are based on tenancy rental agreements. This means that there could be biases in the differences in opportunity costs between land systems. For example, the actual net economic benefits of agricultural land are likely higher than only tenancy rental costs, given that the latter are only part of the costs involved in production^23,96^, implying that our layers likely underestimate foregone economic benefits of agriculture. Users should be aware of this when making comparisons between the opportunity costs of different land types. In general, it is important to critically consider cost data, the associated conservation actions and accompanying uncertainties before it is used in a spatial conservation prioritisation^15,97–99^. Kujala *et al*. (2018) have shown that a further important factor for consideration is the correlation between cost and biodiversity data. If the relationship is positive, cost data inclusion can lead to low priority scores assigned to biodiversity-rich areas, with potentially dire consequences for biodiversity^16^.

Finally, as our opportunity cost estimates are representative of current land systems, rent and prices, our dataset will become progressively less representative over time, as socio-economic and environmental developments influence rent, production and prices. For example, the expected increase in extreme weather events, such as droughts, is likely to impact agricultural and forestry productivity^100,101^. Similarly, increased exposure to flooding can reduce the rental prices in urban lands, and therefore the urban opportunity costs^102^. This, in turn, is likely to affect rent and price values and therefore opportunity costs. We suggest that future efforts could apply our approach to updated data on rent, production (yield) and market prices, potentially including more fine-grained data, in order to update the opportunity cost estimates.

## Supplementary information

Supplementary Information: Opportunity cost estimates for spatial conservation prioritisation across terrestrial Europe

## Data Availability

The custom code used to generate the opportunity cost layers and the standardised layers is available at https://github.com/dcapitelli/Opportunity-Costs-of-Conservation. For some parts, we used ArcGIS with Python 2.7. In the rest of the code we used Python 3.10. QGIS 3.28 was used for layer rendering. The R package ‘stats’ (version 4.3.1) was used for the linear regression modelling.
