# Supplementary material for: Opportunity cost estimates for spatial conservation prioritisation across terrestrial Europe
Source: Sci Data. 2025 Sep 29;12:1583. doi: 10.1038/s41597-025-05837-5 (PMC12480582; doi:10.1038/s41597-025-05837-5)
Supplement: Supplementary file 1 — Supplementary Information: Opportunity cost estimates for spatial conservation prioritisation across terrestrial Europe [file 41597_2025_5837_MOESM1_ESM.pdf]

Opportunity cost estimates for spatial conservation prioritisation across Europe

Supplementary Information

Contents

Tables:

Supplementary Table 1: Overview of land systems by Sandstrom et al. (2023) and corresponding land categories used for the opportunity cost layers. 2
Supplementary Table 2: Input data sources. 3
Supplementary Table 3: Two letter codes with corresponding countries. 5
Supplementary Table 4: MAGNET world regions and constituent countries. 5
Supplementary Table 5: MapSPAM crops mapped onto the crop categories distinguished by FAOSTAT and Eurostat. 6
Supplementary Table 6: Model selection results for the relationships between residential rent and population density. The selected model (lowest AIC) is in bold. 8

Figures:

Supplementary Figure 1: Residential rent linear regression model plots. 8

Supplementary Table 1: Overview of land systems by Sandstrom et al. (2023) and corresponding land categories used for the opportunity cost layers.

**Land class subdivision**	**Land category**							
	Cropland	Grassland	Forestry land	Urban	Wetland	Shrub	Bare land	Water and glacier
Water and Glacier	-	-	-	-	-	-	-	X
Low intensity settlement	-	-	-	X	-	-	-	-
Medium intensity settlement	-	-	-	X	-	-	-	-
High intensity settlement	-	-	-	X	-	-	-	-
Wetlands	-	-	-	-	X	-	-	-
Forest/shrub and cropland mosaic	X	-	X	-	-	X	-	-
Forest/shrub and grassland mosaic	-	X	X	-	-	X	-	-
Low intensity arable cropland	X	-	-	-	-	-	-	-
Medium intensity arable cropland	X	-	-	-	-	-	-	-
High intensity arable cropland	X	-	-	-	-	-	-	-
Permanent cropland	X	-	-	-	-	-	-	-
Low intensity grassland	-	X	-	-	-	-	-	-
medium intensity grassland	-	X	-	-	-	-	-	-
High intensity grassland	-	X	-	-	-	-	-	-
Primary forest	-	-	X	-	-	-	-	-
Nature forest management	-	-	X	-	-	-	-	-
Multifunctional forest	-	-	X	-	-	-	-	-
Intensive forest management	-	-	X	-	-	-	-	-
Plantation forest	-	-	X	-	-	-	-	-
Bare, rock and shrub	-	-	-	-	-	X	X	-

Supplementary Table 2: Input data sources.

Note that source units described here may differ from source units in the methods section (such as €/kg instead of €/tonne here) because of unit harmonisation.

Data type	Name	Description	Source	Resolution	Source unit	Years covered
Price	Agricultural product prices	Producer prices.	FAOSTAT[1]	NUTS 0	USD/tonne	2013-2021
	Agricultural product prices	Data on unit values of agricultural products (crops, vegetables, livestock, etc.) at producer prices expressed in euro per tonne and national currency per tonne.	Eurostat[2]	NUTS 0	Euro/tonne	2010-2021
	Livestock prices	Selling prices of animal products (absolute prices) - annual price	Eurostat[3]	NUTS 0	Euro/tonne	2011-2021
	Timber prices	Export and Import Unit Prices	UNECE/FAO[4]	NUTS 0	USD/1000m^3	2020
Production	Crop production	MapSPAM Version 2.0	International Food Policy Research Institute[5]	5 arc minutes (~10 km^2)	kg/ha	2010
	Forest production	Wood production in European forests	European Forest Institute[6]	1 km^2	m^3/ha/yr	2010
	Livestock production	Gridded Livestock of the World (GLW v4): Global sheep, cattle, and goat distribution in 2015 (5 minutes of arc)	Université Libre de Bruxelles / Food and Agriculture Organization (FAO) [7–9]	5 arc minutes (~10 km^2)	heads/km^2	2010
	Livestock production	Slaughtering in slaughterhouses - annual data	Eurostat[10]	NUTS 0	Thousand tonnes	2018-2021
	Livestock production	Milk collection (all milks) and dairy products obtained – annual data	Eurostat[11]	NUTS 0	Thousand tonnes	2019-2021
Rent	Agricultural land rent		Eurostat[12]; Department of Agriculture, Environment and Rural Affairs (DAERA) of Northern Ireland[13]; Federal Statistical Office of Switzerland[14]; Instituto Nacional de Estatistica, Statistics Portugal[15]; Statistisches Bundesamt (Destatis), Federal Statistical Office of Germany[16]; The Scottish Government, Riaghaltas na h-Alba[17]; Wageningen Economic Research,	NUTS 0, 1, 2, 3, and MAGNET regions	Euro/ha	2009-2021

Data type	Name	Description	Source	Resolution	Source unit	Years covered
			the Joint Research Centre and the Thünen-Institute[18]			
	Forestry economic benefits	Forest rents	World Bank[19]	NUTS 0	% of GDP	2021
	GDP	GDP (current US$)	World Bank[20]	NUTS 0	USD	2021
	Residential rent	Average rent per month in cities by type of dwelling	Eurostat[21]	City	Euro/dwelling type	2020-2021
Municipal statistics	Population	Population per city	Eurostat[22]; UN statistics[23]; Statistical Office of Albania[24]; Statistical Office of Montenegro[25]; Agency of statistics of Bosnia and Herzegovina[26]; Statistical office of Kosovo[27]; Office for National Statistics of the UK[28]; Municipal Office of Belgrade[29]; Wikipedia[30–32]	City	Total population	2011-2017
	Proportion of population	Percentage of population living in each dwelling type	Eurostat[33]	NUTS 0	%	2018-2021
	City area		Albanian Association of Municipalities[34]; Municipal Office of Belgrade[29]; City Population[35]; Eurostat[36]; Republic of Kosovo[37]; Wikipedia[30–32]	City	km^2	2011-2023
	Population	Gridded population	CLUMondo Model[38]	1 km^2	Heads/km^2	2020

Supplementary Table 3: Two letter codes with corresponding countries.

Code	Country	Code	Country	Code	Country
AL	Albania	EL	Greece	PL	Poland
AD	Andorra	HU	Hungary	PT	Portugal
AT	Austria	IE	Ireland	RO	Romania
BE	Belgium	IT	Italy	SM	San Marino
BA	Bosnia and Herzegovina	XK	Kosovo	RS	Serbia
BG	Bulgaria	LI	Liechtenstein	SK	Slovakia
HR	Croatia	LV	Latvia	SI	Slovenia
CY	Cyprus	LT	Lithuania	ES	Spain
CZ	Czechia	LU	Luxembourg	SE	Sweden
DK	Denmark	MT	Malta	CH	Switzerland
EE	Estonia	ME	Montenegro	UK	United Kingdom
FI	Finland	NL	Netherlands	VA	Vatican City
FR	France	MK	North Macedonia		
DE	Germany	NO	Norway		

Supplementary Table 4: MAGNET world regions and constituent countries.

Only European countries are listed.

Region	Countries (ISO codes)
Western Europe	Andorra (AD), Austria (AT), Belgium (BE), Denmark (DK), Finland (FI), France (FR), Germany (DE), Greece (EL), Ireland (IE), Italy (IT), Liechtenstein (LI), Luxembourg (LU), Malta (MT), Netherlands (NL), Norway (NO), Portugal (PT), San Marino (SM), Spain (ES), Sweden (SE), Switzerland (CH), United Kingdom (UK) Vatican City State (VA)
Central Europe	Albania (AL), Bosnia and Herzegovina (BA), Bulgaria (BG), Croatia (HR), Cyprus (CY), Czech Republic (CZ), Estonia (EE), Hungary (HU), Latvia (LV), Lithuania (LT), Macedonia, FYR (MK), Poland (PL), Romania (RO), Serbia and Montenegro (RS & ME), Slovak Republic (SK), Slovenia (SI)

Supplementary Table 5: MapSPAM crops mapped onto the crop categories distinguished by FAOSTAT and Eurostat.

FAOSTAT groups are used to map FAOSTAT crops onto Eurostat crop categories when Eurostat crop categories lack sufficient specificity. ++ refers to aggregated FAOSTAT crop categories.

**MapSPAM**	**FAOSTAT**			**Eurostat**	
	Name	Code	Group	Name	Code
Wheat	Wheat	15	Cereals	Wheat and spelt	01100
Rice	Rice	27	Cereals	Rice	01600
Maize	Maize	56	Cereals	grain maize, fodder maize	01500; 03100
Barley	Barley	44	Cereals	Barley	01300
Pearl millet	Millet	79	Cereals	Cereals (including seeds)	01000
Small millet	Millet	79	Cereals	Cereals (including seeds)	01000
Sorghum	Sorghum	83	Cereals	Cereals (including seeds)	01000
Other Cereals	Other Cereals ++	68, 71, 75, 89, 92, 94, 97, 101, 103, 108	Cereals	Oats and summer cereal mixtures	01400
Potato	Potato	116	Roots & Tubers	Potatoes for consumption; Industrial potatoes; Potato seeds; Fodder potatoes [a]	73600; 73700; 73800; 73900
Sweet Potato	Sweet Potato	122	Roots & Tubers	Potatoes for consumption	73600
Yams	Yam	137	Roots & Tubers	Potatoes for consumption	73600
Bean	Beans, Dry	176	Pulses	haricot beans	73500
Chickpea	Chickpea	191	Pulses	dried pulses	73300
Cowpea	Cowpea	195	Pulses	dried pulses	73300
Pigeon Pea	Pigeon Pea	197	Pulses	dried pulses	73300
Lentil	Lentils	201	Pulses	dried pulses	73300
Other Pulses	Broad Beans ++	181, 187, 203, 205, 210, 211	Pulses	dried pulses	73300
Soybean	Soybean	236	Oilcrops	haricot beans	73500
Groundnut	Groundnut, With Shell	242	Oilcrops	Oil seeds and oleaginous fruits (including seeds)	02100
Coconut	Coconut	249	Oilcrops	Oil seeds and oleaginous fruits (including seeds)	02100
Oilpalm	Oil Palm Fruit	254	Oilcrops	Oil seeds and oleaginous fruits (including seeds)	02100
Sunflower	Sunflower Seed	267	Oilcrops	Oil seeds and oleaginous fruits (including seeds)	02100
Rapeseed	Rapeseed, Mustard seed	270, 292	Oilcrops	Oil seeds and oleaginous fruits (including seeds)	02100
Sesame Seed	Sesame Seed	289	Oilcrops	Oil seeds and oleaginous fruits (including seeds)	02100
Other Oil Crops	Olives ++	260, 263, 265, 275, 280, 296, 299, 333, 336, 339	Oilcrops	Olive	06500

MapSPAM	FAOSTAT			Eurostat	
	Name	Code	Group	Name	Code
Sugarcane	Sugar Cane	156	Sugar Crops	Sugar beet	02400
Sugarbeet	Sugarbeet	157	Sugar Crops	Sugar beet	02400
Cotton	Seed Cotton	328	Fibres	Fibre plants	02910
Other Fibre Crops	Other Fibres ++	773,777, 780, 782, 788, 789, 800,	Fibres	Fibre plants	02910
Other Fibre Crops	Other Fibres ++	809, 821	Fibres	Fibre plants	02910
Tea	Tea	667	Stimulates	-	-
Tobacco	Tobacco	826	Stimulates	-	-
Banana	Banana	486	Fruits	-	-
Tropical Fruit	Oranges ++	490, 495, 497, 507, 512, 567, 568, 569, 571, 572, 574, 577, 587, 591, 600, 603	Fruits	sweet oranges; mandarins; lemons; clementines	06210; 06220; 06230; 76200
Temperate Fruit	Apples ++	515, 521, 523, 526, 530, 531, 534, 536, 541, 542, 544, 547, 549, 550, 552, 554, 558, 560, 592, 619	Fruits	desert apples; desert pears; peaches; grapes; tomatoes; apricot; cherries; strawberries; currants; gooseberry	06110; 06120; 06130; 06400; 04120; 75500; 75600; 75900; 76000; 76100
Vegetables	Cabbages And Other Brassicas ++	358, 366, 367, 372, 373, 388, 393, 394, 397, 399, 401, 402, 406, 407, 414, 417, 420, 423, 426, 430, 446, 449, 459, 461, 463	Vegetables	cauliflower; cabbages; fennel; endives; artichokes; courgettes; cucumbers; onions, shallots	04110; 74200; 74500; 74300; 74400; 74800; 74700; 75100
Rest Of Crops	All Individual Other Crops (e.g., Spices, Tree Nuts, Other Sugar Crops, Mate, Rubber)	161,216, 217, 220, 221, 222, 223, 224, 225, 226, 234, 671, 677, 687, 689, 692, 693, 698, 702, 711, 720, 723, 748, 754, 836, 839		walnuts; hazelnuts	75700; 75800

[a] Excluded: "Potatoes (including seeds)

Supplementary Table 6: Model selection results for the relationships between residential rent and population density. The selected model (lowest AIC) is in bold.

Model	*Adjusted R^2*	*F-statistic*	*p value*	*df*	*logLik*	*AIC*	*BIC*	*deviance*	*n*
**LogRent ~ LogPop + EU_region**	0.95	194.86	2.58E-24	4	-0.93	13.86	24.29	2.570391	42
LogRent ~ LogPop * EU_region	0.95	107.94	2.48E-21	7	0.14	17.71	33.35	2.44236	42
LogRent ~ LogPop	0.89	318.09	1.21E-20	1	-19.87	45.75	50.96	6.335749	42
LogRent ~ Pop + EU_region	0.62	17.88	2.98E-08	4	-43.3	98.6	109	19.33596	42
LogRent ~ Pop	0.56	52.56	8.44E-09	1	-48.3	102.6	107.8	24.51108	42
LogRent ~ Pop * EU_region	0.59	9.46	1.83E-06	7	-43.2	104.4	120.1	19.24794	42
Rent ~ LogPop + EU_region	0.77	35.26	3.73E-12	4	-524.2	1060.5	1070.9	1.71E+11	42
Rent ~ LogPop * EU_region	0.77	20.32	1.97E-10	7	-522.7	1063.3	1079	1.58E+11	42
Rent ~ LogPop	0.62	67.31	4.18E-10	1	-536.5	1079	1084.2	3.06E+11	42

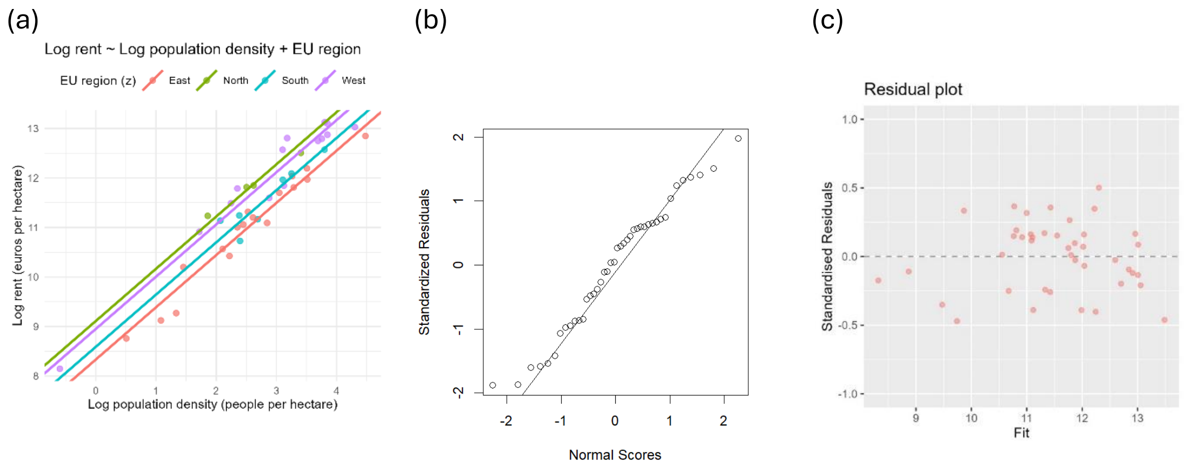

Supplementary Figure 1: Residential rent linear regression model plots.

(a) Linear regression model relationship of log transformed rent and log transformed population density. The model is specified as follows: log(Rent per hectare) = 8.33 + log(Population per hectare) + 0.7781EU region$_{North}$ + 0.2569EU region$_{South}$ + 0.6175EU region$_{West}$ where the East region serves as the reference category (and intercept of 8.33). (b) Quantile-quantile plot of the linear regression model. (c) Residual plot of linear regression model.

References:

1. FAOSTAT. Producer Prices. [Data set]. (2023).
2. Unit values at producer prices. Eurostat https://doi.org/10.2908/AACT_UV02 (2020).
3. Selling prices of animal products (absolute prices) - annual price (from 2000 onwards). Eurostat https://doi.org/10.2908/APRI_AP_ANOUTA (2023).
4. Export and Import Unit Prices (1964-2020). UNECE/FAO https://unece.org/forests/prices (2023).
5. Yu, Q. *et al.* A cultivated planet in 2010 – Part 2: The global gridded agricultural-production maps. *Earth Syst. Sci. Data* **12**, 3545–3572 (2020).
6. Verkerk, P. J. *et al.* Data from: Mapping wood production in European forests. 169784641 bytes Dryad https://doi.org/10.5061/DRYAD.MK067 (2015).
7. Gilbert, M. *et al.* Global cattle distribution in 2015 (5 minutes of arc). Harvard Dataverse https://doi.org/10.7910/DVN/LHBICE (2022).
8. Gilbert, M. *et al.* Global sheep distribution in 2015 (5 minutes of arc). Harvard Dataverse https://doi.org/10.7910/DVN/VZOYHM (2022).
9. Gilbert, M. *et al.* Global goats distribution in 2015 (5 minutes of arc). Harvard Dataverse https://doi.org/10.7910/DVN/YYG6ET (2022).
10. Slaughtering in slaughterhouses - annual data. Eurostat https://doi.org/10.2908/APRO_MT_PANN (2023).
11. Milk collection (all milks) and dairy products obtained – annual data. Eurostat https://doi.org/10.2908/APRO_MK_POBTA (2023).
12. Agricultural land renting prices for one year by region. Eurostat https://doi.org/10.2908/APRI_LRNT (2023).
13. Department of Agriculture, Environment and Rural Affairs (DAERA) of Northern Ireland. *Farm Business Data*. https://www.daera-ni.gov.uk/sites/default/files/publications/daera/Farm%20Business%20Data%202022.pdf (2022).
14. Comptes régionaux de l'agriculture, par canton et par région agricole: de la production au revenu. Federal Statistical Office of Switzerland https://www.bfs.admin.ch/bfs/fr/home/statistiques/catalogues-banques-donnees/donnees.assetdetail.23425351.html (2022).
15. Contas económicas da agricultura regionais (preços correntes; anual) - Portugal. Instituto Nacional de Estatistica, Statistics Portugal https://www.ine.pt/xportal/xmain?xpid=INE&xpgid=ine_cnacionais2010b2016&contexto=cs&selTab=tab3&perfil=392023991&INST=391970297 (2022).
16. Land- und Forstwirtschaft, Fischerei: Eigentums- und Pachtverhältnisse Landwirtschaftszählung. Statistisches Bundesamt (Destatis), Federal Statistical Office of Germany https://www.destatis.de/DE/Themen/Branchen-Unternehmen/Landwirtschaft-Forstwirtschaft-Fischerei/Landwirtschaftliche-Betriebe/Publikationen/Downloads-Landwirtschaftliche-Betriebe/eigentums-pachtverhaeltnisse-2030216209004.pdf?__blob=publicationFile (2021).
17. Tenanted Agricultural Land in Scotland. The Scottish Government, Riaghaltas na h-Alba https://www.gov.scot/binaries/content/documents/govscot/publications/statistics/2017/04/tenanted-agricultural-land-scotland-2016-17/documents/00517139-pdf/00517139-pdf/govscot%3Adocument/00517139.pdf#:~:text=The%20average%20rent%20in%202016%2F17%20was%20estimated%20as,LFA%20holdings%20and%20five%20per%20cent%20for%20non-LFA. (2017).
18. Doelman, J. C. *et al.* Afforestation for climate change mitigation: Potentials, risks and trade-offs. *Global Change Biology* **26**, 1576–1591 (2020).
19. Forest rents (% of GDP). World Bank https://data.worldbank.org/indicator/ny.gdp.frst.rt.zs (2022).

20. GDP (current US$). World Bank https://data.worldbank.org/indicator/NY.GDP.MKTP.CD (2023).
21. Average rent per month in cities by type of dwelling. Eurostat https://doi.org/10.2908/PRC_COLC_RENTS (2023).
22. Regions and Cities Illustrated (RCI). Eurostat https://ec.europa.eu/eurostat/cache/RCI/#?vis=nuts2.labourmarket&lang=en (2023).
23. City population by sex, city and city type. United Nations Statistics Division https://data.un.org/Data.aspx?d=POP&f=tableCode%3A240 (2023).
24. Population and Housing Census. Instituti I Statistikës https://web.archive.org/web/20170823071731/http://www.instat.gov.al/media/195838/11__tirane.pdf (2013).
25. Census 2011 data - Settlements. Statistical Office of Montenegro http://www.monstat.org/eng/page.php?id=395&pageid=57 (2011).
26. Census of Population, Households and Dwellings in Bosnia and Herzegovina. Agency of statistics of Bosnia and Herzegovina https://popis.gov.ba/popis2013/doc/RezultatiPopisa_BS.pdf (2013).
27. *2011 Census of Population, Households and Housing in Kosovo – Final Results: Demographic Data by Municipality*. https://web.archive.org/web/20160304081103/http://ask.rks-gov.net/rekos2011/repository/docs/Te%20dhenat%20kryesore%20demografike%20sipas%20komunave.pdf (2016).
28. 2011 UK censuses. Office for National Statistics of the UK https://www.ons.gov.uk/census/2011census/2011ukcensuses (2011).
29. City of Belgrade. Territory. https://www.beograd.rs/index.php?lang=cir&kat=beoinfo&sub=201197%3f (2023).
30. Podgorica. *Wikipedia* https://en.wikipedia.org/wiki/Podgorica (2023).
31. Sarajevo. *Wikipedia* https://en.wikipedia.org/wiki/Sarajevo (2023).
32. Skopje. *Wikipedia* https://en.wikipedia.org/wiki/Skopje (2023).
33. Distribution of population by degree of urbanisation, dwelling type and income group - EU-SILC survey. Eurostat https://doi.org/10.2908/ILC_LVHO01 (2023).
34. Albanian Association of Municipalities. *Bashkia Tirana*. https://web.archive.org/web/20201012193031/https://aam.org.al/en/bashkia-tirane/ (2011).
35. Culham (Parish, United Kingdom) - Population Statistics, Charts, Map and Location. https://www.citypopulation.de/en/uk/southeastengland/admin/south_oxfordshire/E04012476__culham/ (2023).
36. Urban Audit 2021. Eurostat https://ec.europa.eu/eurostat/web/gisco/geodata/reference-data/administrative-units-statistical-units/urban-audit (2021).
37. Republic of Kosovo. *Komisioni për majten e territorit të Republikës së Kosovës*. https://web.archive.org/web/20200922113615/https://kryeministri-ks.net/wp-content/uploads/2018/03/Raport_Faktografik_Matja_e_territorit_te_Republikes_se_Kosoves_032017.pdf (2017).
38. Verburg, P. CLUMondo Model. DataverseNL https://doi.org/10.34894/XZCPQY (2024).
